# Opposition to Pharmacist Contraception Services: Evidence for Rebuttal

**DOI:** 10.3390/pharmacy8040176

**Published:** 2020-09-23

**Authors:** Madeline Mitchell, Courtney Stauffenberg, Veronica Vernon, Cortney M. Mospan, Allie Jo Shipman, Sally Rafie

**Affiliations:** 1 College of Pharmacy and Health Sciences, Butler University, Indianapolis, IN 46208, USA; mmmitch1@butler.edu (M.M.); cstauffe@butler.edu (C.S.); 2 Department of Pharmacy Practice, College of Pharmacy and Health Sciences, Butler University, Indianapolis, IN 46208, USA; vvernon@butler.edu; 3 School of Pharmacy, Wingate University, Wingate, NC 28174, USA; c.mospan@wingate.edu; 4 National Association of State Pharmacy Associations, North Chesterfield, VA 23235, USA; ajshipman@naspa.us; 5 Birth Control Pharmacist, San Diego, CA 92122, USA

**Keywords:** contraception, birth control, pharmacist, public health services, contraception access, pharmacist prescribing, contraception services, birth control pharmacy

## Abstract

Pharmacist contraception services are growing across the United States. Several states have authorized pharmacists to prescribe contraception, and the interest in other states continues to grow. Opposition to these practices exists and centers on discussions related to safety, training, cost, and fragmentation of care. We review these arguments and provide evidence refuting these concerns. Pharmacist-prescribed contraception increases access to care, and patients express interest in utilizing this service at the pharmacy. Pharmacists follow evidence-based recommendations. Counseling on preventative services and referral to other providers is part of contraception care by pharmacists. Training programs have been developed to equip both pharmacy students and pharmacists with the knowledge, skills, and tools needed to successfully provide these services. This article can serve as a guide for pharmacists and advocates when discussing pharmacist-prescribed contraception with policymakers, patients, and other healthcare professionals.

## 1. Introduction

Since 2013, 13 states and jurisdictions in the United States (U.S.) have been successful in passing legislation allowing for pharmacist-prescribed contraception, also known as pharmacy access to contraception. These include California, Colorado, the District of Columbia, Hawaii, Idaho, Maryland, Minnesota, New Hampshire, New Mexico, Oregon, Utah, Virginia, and West Virginia. In 2019 alone, 15 states introduced legislation to allow for pharmacist-prescribed contraception; however, only 1 state was successful in their efforts to pass pharmacist-prescribed contraception [1]. In total, at least 31 states have considered legislation allowing for pharmacist-prescribed contraception services. With only 25% of states currently having provisions for autonomous pharmacist-prescribed contraception, efforts to expand patient access to contraception could be significantly hampered [2]. Further expansion of pharmacist-prescribed contraception in other states could provide a route to help meet the Healthy People 2030 objectives related to preventing unintended pregnancy and increased contraceptive use [3]. One-third of U.S. adults have reported difficulty accessing contraception, but one of the least common barriers is access to a pharmacy [4].

Inaccurate and ill-informed policies are often cited by opponents of pharmacist-prescribed contraception. A recent example is an article in the Healthcare Ethics Committee Forum (HEC Forum), which described pharmacist-prescribed contraception as a “band-aid” [5]. Another objection to pharmacist-prescribed contraception has been voiced by certain physician groups, as this is seen as an expansion of the pharmacist’s scope of practice. The American Medical Association opposes independent prescribing by pharmacists “without appropriate physician supervision” [6]. We advocate for evidence-based policies to expand patient access to contraception services. In this commentary, we will lay out commonly encountered opposition arguments from states that have worked on pharmacist-prescribed contraception efforts, many of which often lack evidence to support their argument. A team of experts in pharmacy-based health policy and advocacy built consensus on the commonly encountered arguments, based on experience working to advance pharmacist-prescribed contraception. Evidence for rebuttal was based on a literature search to identify supporting articles for pharmacist-prescribed contraception. The arguments addressed include (1) safety concerns, (2) patients foregoing health screenings, (3) limited long-acting reversible contraception access, (4) availability of services and concerns for pharmacists’ refusal, (5) pharmacist training, (6) patient utilization, (7) cost, (8) over-the-counter access, and (9) the support of other healthcare providers. For these commonly encountered opposition arguments, we will provide evidence and rationale that pharmacists and other patient advocates can utilize to alleviate these concerns.

## 2. Evidence against Opposition to Pharmacist Contraception Services

### 2.1. Safety

One argument against pharmacist-prescribed contraception is the safety of the process, as hormonal contraception can lead to adverse effects. According to the Center for Disease Control and Prevention (CDC) U.S. Special Practice Recommendations for Contraceptive Use (U.S. SPR), the only examination or test needed prior to initiating combined hormonal contraceptives (CHC) is blood pressure assessment. Progestin-only pills (POP) do not have any requirements, thoughit may be helpful to measure baseline weight and body mass index (BMI) for both CHC and POP methods [7]. Pharmacists are trained to perform blood pressure assessments. A 2019 study found that 98.3% of pharmacists surveyed were comfortable measuring blood pressure [8]. Additionally, it is recommended that providers identify specific medical conditions or characteristics listed in the U.S. Medical Eligibility Criteria for Contraceptive Use (U.S. MEC) [9]. This can be achieved with a simple screening questionnaire that patients complete with their health history information prior to or while talking with their provider about methods of contraception, similar to immunization screening questionnaires.

When patients have contraindications to forms of pharmacist-prescribed contraception, referral to another healthcare provider is indicated. After a simulated contraception activity, 100% of pharmacy students reported feeling confident in their ability to refer patients to a physician when necessary [10]. Pharmacists refer patients for necessary health screenings and contraindications to hormonal contraception [11]. Among patients seeking pharmacist-prescribed contraception in California and Oregon for hormonal contraception, 7% were referred appropriately to a primary care provider based on the U.S. MEC. Elevated blood pressure was the main referral indication [12].

A study including the Oregon Medicaid population concluded that pharmacists are safely following prescribing protocols and identifying contraindications to contraception. Of the patients who received contraception, 5% had a U.S. MEC Category 3 or 4 medical condition that was not identified during pharmacist screening. This frequency of error is similar to that of clinicians’ (5% to 8%) seen in a 2011 study of contraception contraindications [13,14]. A study of telehealth providers found that 7% of appointments were noncompliant with the U.S. MEC [15]. Telehealth has grown exponentially in the past 10 years, giving patients another method for accessing contraceptive care. These programs utilize a questionnaire to determine a patient’s medical eligibility similar to that used by pharmacists when prescribing contraception. Physicians, nurse practitioners, and physician assistants prescribe contraception for patients who utilize these services. The relatively low error rate of all three access models demonstrates safety for patients with or without a physician.

Concerns are often raised about pharmacists not having access to patients’ full medical records and the accuracy of patient’s filling out medical questionnares to determine contraception eligibility. Whenpatient’s responses to screeningquestionnaires were compared with the documentation of their healthcare providers, there was an overall agreement of 96%. Interestingly, patients tended to be more likely to report contraindications such as smoking, irregular bleeding, and possible pregnancy than their providers [11]. This shows that patientsare accurately and honestly filling out questionnaires provided by pharmacists, and these questionnaies adequately identify medical eligibility criteria. These formscan provide the necessary information for a pharmacist to determine patients’ eligilibity for contraception.

### 2.2. Patients Will Forego Recommended Health Screenings

Access to pharmacist-prescribed contraception has raised concerns that patients will forego necessary health screenings. Table 1 summarizes recommendations by expert organizations regarding women’s health screenings. The frequency of screening is often misunderstood and places a barrier to contraception access. The American College of Obstetricians and Gynecologists (ACOG) states, “pelvic and breast exams, cervical cancer screening, and sexually transmitted infections (STI) screening are not required before initiating hormonal contraception and should not be used as reasons to deny access to hormonal contraception” [16]. Table 1 includes recommendations for well-woman health screenings.

In a study that compared women who utilized a family planning clinic versus those who bought over-the-counter (OTC) oral contraceptives in Mexico, most patients utilizing OTC contraception had received a Papanicolaou (Pap) test in the last 3 years (90.8%), as well as a pelvic exam (88.5%), clinical breast exam (88.9%), and STI screening (71.7%) [23]. This observed rate for receiving a Pap test is higher than the most recent national average from 2015 among women 21–65 years old (81%) [24]. STI screening had the lowest rate among OTC users, but in comparison, a 5-year study published in 2010 found that chlamydia screenings over 5 years only occurred in 26% of patients [25]. This evidence shows that more accessible models are not steering patients away from preventative health screenings. Additionally, data from California and Oregon showed that 89% of users who utilized pharmacy contraception services had visited their primary care provider within the last year [12].

Another medical area resulting in a concern for patients foregoing regular screening is telehealth. These programs are comparable to pharmacy access, as neither require a face-to-face visit with a physician, except telehealth is already implemented and widely utilized. It does not involve a blood pressure measurement. Although the concern for foregoing regular health screenings is valid, pharmacists prescribing contraception present an opportunity to educate patients on proper screening at every visit and refer to a provider when necessary.

### 2.3. Long-Acting Reversible Contraception Access

In the United States, pharmacist-prescribed contraception does not include long-acting reversible contraception (LARC), which some opponents cite as a disadvantage that may deter the use of LARC methods. Pharmacists provide comprehensive methods counseling and are well positioned to refer patients and educate them on the benefits of LARC, making this an asset to pharmacist-prescribed contraceptive services rather than a drawback. In a study in the United Kingdom, the number of women receiving a copper intrauterine device (IUD) for emergency contraception increased by three-fold after counseling and referral by a community pharmacist [26]. Pharmacists are able to educate patients on all forms of LARC and make necessary referrals for placement.

Many physicians and other contraception providers do not provide LARC services, requiring them to refer patients for this care as well. Although 80–90% of obstetrics–gynecology providers report placing LARC, availability by other specialties is significantly lower. Among internal medicine and pediatric physicians, this decreases to 26% [27,28]. In the American Board of Family Medicine 2019 residency graduate follow-up survey, only 45% of physicians reported currently practicing IUD insertion and removal and 48% reported current practices using subdermal implant contraception [29]. The growing area of telecontraception has also been studied, and it was found that LARC was only mentioned as a contraception option to patients by two of nine companies studied [15]. Referrals to the necessary healthcare providers for LARC are essential and not limited to pharmacist-prescribed contraception services.

While LARC methods are highly effective and safe, a majority of patients continue to choose oral contraception over other non-surgical methods [30]. Patients most commonly choose short-acting methods due to ease of access, low cost, privacy, and freedom to stop use without involving a healthcare provider [31]. For people who prefer short-acting methods, pharmacists can meet their desire for improved access and low costs. Pharmacists can continue to educate on the safety and effectiveness of LARC and refer when appropriate, while providing short-acting contraceptives to patients with those preferences.

### 2.4. Availability and Pharmacist Adoption

Concerns have been raised that pharmacists will not engage in contraception prescribing even if policy allows. Pharmacists have been interested in the ability to provide direct access to hormonal contraception for many years. In 2009, 85% of pharmacists in a nationwide survey expressed interest in prescribing hormonal contraception [32]. One year after implementing legislation in Oregon, 63% of zip codes in Oregon had a pharmacist certified to provide contraception services [33]. In a two-year period in Oregon, 162 pharmacists prescribed contraception for 367 patients on Medicaid. This resulted in over 1300 claims for contraception. Within the first four months of implementation of the statewide protocol, there were an average of 40 Medicaid claims per month for pharmacist-prescribed contraception. This grew to 60 claims per month by month seven. Pharmacists prescribed 10% of all transdermal and oral contraception products for Medicaid patients [13]. Data from Oregon and New Mexico reveal that these services are provided in both rural and urban settings [34]. Nearly 400 pharmacists employed by one supermarket pharmacy chain were prescribing hormonal contraception with an average of nearly five prescriptions per pharmacist in a six-month period [12]. Prevalent evidence exists to support pharmacists’ interests in contraception prescribing, as well as their commitment to do so after states have passed the necessary legislation.

Some of the main barriers pharmacists experience concerning their participitation in prescribing contraception include training, payment for pharmacist services by health plans, and workflow [35]. Several of these factors have been addressed in current protocols or additional efforts by states have been made to ensure pharmacists are recognized as healthcare providers. Training programs have been included in several state protocols to increase pharmacist engagement and participation, with pharmacists feeling increased comfort with prescribing after their training sessions (*p* < 0.004) [36].It is important to write policies that are evidence-based and allow flexibility based on the location of the pharmacist. Use of a mandatory reporting form or rigid algorithm may lead to a lack of uptake by all pharmacy locations. Avoiding age restrictions, contraception method restrictions, use of a mandatory intake form or documentation system, and other unecessary elements will allow for broader uptake and implementation of pharmacy contraception services.

Pharmacies generally offer extended hours and do not require an appointment for contraception. Nationwide, over 3000 pharmacies are currently offering these services [37]. This number continues to grow and contraception access and availability will increase as more states implement policies allowing pharmacists the authority to prescribe contraception.

### 2.5. Pharmacist Training

A lack of pharmacist training in prescribing contraception has been cited as another argument. Pharmacists are highly trained and serve as the medication experts on the healthcare team. The entry level degree is the Doctor of Pharmacy degree and students learn how to take patient histories and perform health screenings, study components of medical diagnoses, and learn to develop assessments and plans for patients that include nonpharmacological and pharmacological treatments. Contraception education is provided in pharmacy schools and as continuing education for pharmacists. In a simulation-based contraception prescribing activity, the majority of pharmacy students (88.9%) found the prescribing protocol simple to follow. Over half of the students (55.6%) did not find the process of prescribing contraception challenging. After completion of the activity, 88.9% of pharmacy students felt confident prescribing contraception if state law were to allow this service [9].

Among a survey of community pharmacists in the United States, 77% agreed that pharmacists are well-trained to provide contraception. In a survey designed to assess pharmacists’ readiness to prescribe contraception, over half (57%) of pharmacists, who by state law cannot prescribe contraception, still felt adequately trained and competent in providing contraception services [38]. Interestingly, the self-perception of participants practicing in states that do allow pharmacists to prescribe contraception was no different than the self-perception of participants practicing in states that do not allow pharmacists to prescribe contraception (*p* = 0.239) [39].

In states with standing orders or statewide protocols for pharmacist-prescribed contraception, all require completion of a training program. California accepts in-state curriculum-based training programs [40]. Existing literature has highlighted gaps in contraception education among medical schools and training programs for nurse practitioners [41,42]. Pharmacists are well-equipped to provide this service, and several training programs exist for additional education opportunites when needed.

### 2.6. Patient Utilization

The question of whether patients will want to use the pharmacy for contraception has been raised. Several studies have found that patients appreciate the convenience and accessibility of pharmacies, which drives pharmacy utilization to access contraception services [43,44,45,46]. In a California study, 87% of patients reported they were likely to return to the pharmacy, 90% would refer a friend, and 95% reported overall satisfaction with pharmacist-prescribed contraception [46]. Among a survey of Michigan college students, 46% responded they were extremely likely and 26% were moderately likely to obtain contraception from a pharmacist. The main reasons cited for this were convenience, less time than a traditional office visit, and easier adherence [47]. In a national telephone survey of adult women, 68% of them stated they were likely to use contraception services provided by pharmacists. Uninsured and low-income women were more likely to report as potential users. In the same survey, 66% of women already on hormonal contraception were likely to use pharmacist-provided services and 42% of women not on any form of contraception reported they were likely to begin using hormonal contraception if provided by pharmacists [45]. Patients in four states were surveyed regarding their reasons for utilizing pharmacist-prescribed contraception. The two most common answers were that an appointment was not required or their previous prescription lapsed [48].

Qualitative studies have shown 97% of interviewed adolesents from California and 56% of interviewed adolescents from Indiana are supportive and interested in contraception prescribed by pharmacists. Both groups of study participants appreciated the convenience and accessibility of receiving care in a pharmacy setting. Adolescents have also noted in qualitative studies that pharmacists are likely more educated on the various forms of contraception [43,44].

The privacy offered in a pharmacy setting is often stated as a barrier to patient utilization; however, many pharmacies have adapted to allow for private patient counseling spaces. It is essential that the pharmacist and patient have access to a private area. Pharmacies without access to private patient counseling areas may require structural changes to address privacy concerns. Despite potential concerns for privacy, patients are utilizing pharmacist-prescribed contraception services in states with legislation in place.

In 2019, one supermarket-based pharmacy chain in California and Oregon provided contraception services to over 2000 patients within a 6.5-month time period [12]. In Oregon, after two years of statewide protocol implementation, 10% of Medicaid patients received a contraceptive prescription through a pharmacist, with most patients being new users [9]. After recieving 12 months of pharmacist prescribed-contraception services, 96.8% of patients (ages 13–55 years of age) felt comfortable with continuing their contraceptive services with a pharmacist [11]. Patients have been proven to be comfortable, as well as satisfied with their participation in the services. At a 1-month follow-up of patients receiving pharmacist-prescribed contraception, 97.7% reported feeling satisfied with the service and 97.1% stated they would refer the service to their friends [11]. In addition to all the qualitative benefits, there has also been an improvement in health outcomes. A cost-effective analysis of the services offered in Oregon found 158 quality-of-life-years were gained per 198,000 women [49].

### 2.7. Cost

Out-of-pocket cost is a major concern for patients, but states with active protocols for pharmacist-prescribed contraception have continued to provide cost-effective care while also gathering the support of public insurance and commercial insurance companies. Medication cost is covered by insurance, regardless of the prescriber. Insurance coverage for the patient visits is a concern, as pharmacists are not able to bill for medical services in all states. California addressed this through successful legislation in 2016, which requires the state Medicaid program to pay for pharmacist contraception services [50]. The District of Columbia, Maryland, and Oregon included coverage of pharmacist services in their bills [1,51]. Other states, such as Washington, New Mexico, Virginia, and West Virginia, have passed payment parity legislation, which requires insurers to pay pharmacists for services provided if the insurer pays other healthcare providers for the service [52,53,54,55]. The cost of contraception when prescribed by a pharmacist was covered by insurance in over 50% of pharmacies in a study of contraception availability in New Mexico and Oregon [34].

Additionally, insurance coverage for dispensing 12 months of contraception at once should be available to all patients. Patients who are able to receive the entire year of contraception in one fill are more likely to continue their method [56]. Pharmacists more frequently prescribed a 6-month or more supply of contraception at one time compared to other providers in one study [57]. Pharmacist-prescribed contraception does significantly positively impact access to contraception care.

While insurance coverage is a coveted factor of contraception services for many, research indicates it may not be necessary for all at-risk groups. An adolescent-aged qualitative study participant stated other adolescents may prefer a cash option to receive contraception services [43]. Pharmacist-prescribed contraception is cost effective and reduces healthcare costs. In 2019, pharmacist-prescribed contraception in Oregon was estimated to prevent 51 unintended pregnancies and saved $1.6 million dollars at only 24 months post-implementation [49]. The cost savings of hormonal contraception access in pharmacies is expected to greatly exceed the cost expenses, and as data emerges, pharmacists can approach insurance companies to expand coverage of these services.

### 2.8. Over-the-Counter Access

Several practitioners and physicians associations argue that the most effective way to offer contraceptive services to patients is over-the-counter (OTC). ACOG cites that robust literature demonstrates the ability of women to self-screen for contraindications to hormonal contraception, based on the U.S. MEC [16]. While OTC access is an important goal, pharmacist prescribing is seen as a parallel effort to provide patients options. There are several barriers that may prevent OTC contraception from being effective and must be addressed.

The biggest concern is loss of insurance coverage if contraception becomes OTC. Patients in lower socioeconomic classes are at higher risk and may not be able to afford contraception without insurance coverage. This notion was confirmed when the biggest concern among women with public insurance was potential cost [58]. Insurance coverage would need to be mandated in order to maximally increase access.

Another concern is the immediacy of access once one product is approved. Creating an OTC product or switching from prescription to OTC is a difficult and lengthy process. Each OTC product switch is handled separately by the Food and Drug Administration (FDA) and may take longer than the 10-month targeted timeframe [59]. This means the switch to OTC contraception will not be immediate, nor will it apply to all forms of self-administered hormonal contraception. Instead, each pharmaceutical company would have to file applications and be approved for each product separately. In contrast, after pharmacist-prescribed contraception legislation was passed in Oregon, rules were enacted and pharmacists were prescribing within five months [60]. Pharmacist-prescribed services could be a bridge to the OTC model and facilitate increased access until a more inclusive approach is solidified. Pharmacist-prescribed contraception can also continue to be an option for patients who desire the service even after OTC contraception is available.

With the push for OTC contraception access, some argue that pharmacist-prescribed contraception is only a temporary solution, or in some cases, replaces one barrier with another. Removal of all barriers for contraception is the ultimate goal, as patients have proven to be competent at self-screening and identifying the necessity of provider consultation [11]. In addition to pharmacy access to contraception, this includes OTC access to self-administered hormonal contraception, ensuring insurance coverage for all methods, removal of age restrictions for contraception access, expanding access to care through telehealth and other modalities, and promoting comprehensive reproductive health education for all providers.

### 2.9. Other Healthcare Provider Support

Some have questioned the support of physicians regarding pharmacist-prescribed contraception. The American College of Obstetricians and Gynecologists (ACOG) and American Academy of Family Physicians have issued policy statements supporting OTC access to contraception [16,61]. In October 2019, the ACOG updated their statement to read “pharmacist-provided contraception may be a necessary immediate step to increase access to contraception” [16]. Physicians have supported legislation for pharmacist-prescribed contraception; the Oregon representative who authored the successful legislation in 2015 is a physician [62]. Physicians have testified in support of state legislation, in addition to other healthcare providers. The author of the successful California legislation was an optometrist.

Physicians, nurse practitioners, nurse midwives, and physician assistants in California supported pharmacist-prescribed contraception in one study. Compared to prescription-only, behind-the-counter, and OTC, pharmacist-prescribed contraception was the most supported method [63]. In a national survey of providers, 76% of physician respondents were in favor of pharmacist-prescribed hormonal contraception [64]. More physicians supported pharmacist-initiated contraception over behind-the-counter or over-the-counter access. A majority of providers also felt comfortable referring patients to a pharmacist. Pharmacists are essential members of the healthcare team and communicate regularly with other providers. In all states with pharmacist-prescribed contraception, the pharmacist notifies the patient’s primary care provider if contraception is prescribed or refers the patient for further care.

## 3. Next Steps for Further Research

Although existing literature overwhelmingly supports pharmacist-prescribed contraception, further data from states implementing this service is desired. Additional research on the relationships of cost and health plan coverage, policy approaches to pharmacist-prescribed contraception (i.e., collaborative practice agreement, standing order, statewide protocol, independent prescriptive authority), and outcomes would further inform future policy efforts. Data from other countries could also aid in policy efforts on an international scale, as pharmacists in four provinces in Canada can prescribe contraception [65]. A case has also been made in the United Kingdom to allow self-administered hormonal contraception to be available directly from pharmacists [66].

We encourage pharmacists across the country to engage policymakers in discussions about contraceptive access. In states that permit pharmacist-prescribed contraception, removing barriers to insurance coverage and implementation in rural and underserved communities is vital. For those in states without this service, it is important to engage key stakeholders, such as health insurance plans, physicians and other healthcare providers, pharmacist associations, and public health and patient advocates. Focusing on evidence and avoiding emotional dialogue and unproven arguments will aid in discussions. Providing examples of data and outcomes from other states will help further make the case for pharmacist-prescribed contraception. Consideration must be given to payment mechanisms for patients. Additionally, policies should allow for pharmacists to utilize the full extent of their knowledge and training and avoid unnecessary restrictions on pharmacists. Policymakers should also permit flexibility with regards to documentation, as pharmacist practice locations vary in the type of electronic health record and/or pharmacy system utilized.

## 4. Conclusions

Opposition arguments to pharmacist-prescribed contraception are often not evidence-based. It is important to focus on evidence related to both contraception and pharmacist contraception care. Pharmacists are highly accessible and trained healthcare providers. They are uniquely positioned to reach patients who may not have a cost-effective or accessible method for obtaining contraception. Patients also prefer the convenience of pharmacist-prescribed contraception. Pharmacists can refer patients for follow-up care, such as LARC methods and other routine health screenings. Utilizing all available members of the healthcare team to improve contraceptive access and care should be the center of these discussions. Patients benefit when all healthcare providers are permitted to practice at the top of their education and training. We encourage stakeholders to collaborate on pharmacist-prescribed contraception policies with state pharmacy associations and Birth Control Pharmacist.

## Figures and Tables

**Table 1:** Recommended well-woman health screenings [17,18,19,20,21,22].

	Pap Test	Pelvic Exam	Clinical Breast Exam	Sexually Transmitted Infections
Screening Recommendation	Every 3 years for those aged 21–65; every 5 years starting at age 30 with hrHPV	Shared decision-making with patient; perform when indicated by medical necessity	Every 1–2 years for women aged 25–39 years old; annually for women over 40	Test based on age and risk factors ^1^
Supporting Organizations	ACOG, ASCCP, SGO	ACOG, USPSTF	ACOG, NCCN	CDC

Abbreviations: hrHPV = high-risk human papillomavirus, ACOG = American College of Obstetricians and Gynecologists, ASCCP = American Society for Colposcopy and Cervical Pathology, SGO = Society of Gynecologic Oncology, USPSTF = United States Preventative Services Task Force, NCCN = National Comprehensive Cancer Network, CDC = Centers for Disease Control and Prevention. ^1^ Chlamydia and gonorrhea for those less than 25 years old and sexually active or 25 and older with risk factors; Human immunodeficiency virus (HIV) for all women ages 13–64 and those who desire sexually transmitted infection (STI) screening.
